# Simultaneous Measurement of Peripheral Ocular Aberrations Using a Virtual Multi-Eccentric Hartmann–Shack Aberrometer

**DOI:** 10.3390/s26144419

**Published:** 2026-07-12

**Authors:** Jennyfer Morales-Marín, Andrés Osorno-Quiroz, Walter Torres-Sepúlveda, Alejandro Mira-Agudelo

**Affiliations:** 1 Grupo de Óptica y Fotónica, Instituto de Física, Facultad de Ciencias Exactas y Naturales, Universidad de Antioquia UdeA, Calle 70 No. 52-21, Medellín 050010, Colombia; alejandro.mira@udea.edu.co; 2 Área de Ciencias Básicas, Grupo de Investigación en Innovación Digital y Desarrollo Social (INDDES), Facultad de Ciencias y Humanidades, Institución Universitaria Digital de Antioquia, Cra. 55 No. 42-90, Medellín 050015, Colombiawalter.torres@prof.iudigital.edu.co (W.T.-S.)

**Keywords:** wavefront reconstruction, multi-eccentric Hartmann–Shack aberrometer, simultaneous peripheral measurement, peripheral ocular aberrations, convolutional neural network

## Abstract

**Highlights:**

**What are the main findings?**

- A computational framework for simultaneous multi-eccentric Hartmann–Shack wavefront sensing from a single acquisition was developed and validated.
- The proposed ResNet-SimAM reconstruction method outperformed the conventional centroid-based approach under realistic image degradation conditions.

**What are the implications of the main findings?**

- The proposed framework enables objective evaluation of reconstruction algorithms for peripheral wavefront sensing before experimental implementation.
- Simultaneous multi-eccentric wavefront sensing may facilitate faster characterization of peripheral ocular aberrations for future ophthalmic applications.

**Abstract:**

We present a computational proof of concept, inspired by a recent patent developed by Nikolai Suchkov and Siegfried Wahl (European Patent Office EP4179957A1), of a virtual multi-eccentric Hartmann–Shack (HS) aberrometer for simultaneous measurement of peripheral ocular aberrations. The simulated system records nine wavefronts distributed within a region of approximately 20° around the fovea and is evaluated under ideal and realistic conditions, including speckle and illumination inhomogeneities in HS sensor images. Aberrations are quantified by two approaches: a traditional centroid-based method and a custom-made ResNet convolutional neural network (CNN) trained on simulated HS images. Results show that speckle markedly degrades traditional reconstructions for eccentric points, whereas the CNN (trained with speckled data) exhibits greater robustness and accuracy across all nine wavefronts. This model serves as a proof of concept toward a physical multi-eccentric aberrometer that acquires nine wavefronts in a single shot, thereby eliminating sequential point-by-point acquisition, reducing measurement time, and limiting motion-induced variability between measurements. The approach has potential applications in evaluating ocular treatments and in longitudinal studies of ametropias where peripheral aberrations are implicated.

## 1. Introduction

Research on peripheral vision dates back approximately 90 years. Over this period, subjective and objective methods have been developed to measure aberrations at multiple eccentricities along the visual axis, both horizontally and vertically relative to the fovea. Interest has intensified due to the debate about the association between peripheral retinal image quality and myopia, one of the most common visual disorders worldwide [1].

Evidence remains mixed. Some studies suggest that peripheral image quality contributes to ametropia development, for example, that peripheral hyperopic defocus may stimulate axial elongation of the eye [2,3], whereas others report no significant association between peripheral refraction and myopic progression [4,5,6]. This debate has motivated more precise peripheral aberrometry; however, most measurements are still acquired sequentially and point by point on the retina, an inefficient and often slow procedure [7,8,9,10,11,12,13]. This sequential acquisition makes peripheral measurements particularly sensitive to eye movements, fixation instability, and temporal fluctuations in accommodation, which can introduce inconsistencies between retinal locations. A notable step forward appeared in 2020 with the first simultaneous method, which nonetheless sampled only two retinal locations using two Hartmann–Shack (HS) sensors [14]. Moreover, scaling to additional field points often requires duplicated sensing elements or increasingly complex optical layouts, limiting straightforward implementation. Together, these factors indicate that a truly single-shot Hartmann–Shack system capable of capturing multiple peripheral wavefronts simultaneously using a single sensor remains an open technical challenge.

Here we address this challenge by building on the patent by Nikolai Suchkov and Siegfried Wahl [15] and proposing a computational model of a multi-eccentric HS sensor. This virtual sensor simultaneously samples nine retinal locations distributed within 20° of the fovea, enabling single-shot acquisition of peripheral and near-foveal measurements. We further introduce custom software that identifies and quantifies on- and off-axis aberrations in parallel, reporting independent aberrometric estimates at each location. By design, this framework aims to provide a real-time overview of optical quality across multiple retinal points while reducing sensitivity to between-location variability inherent to sequential acquisition.

The software integrates parallel processing and convolutional neural networks (CNNs) with wavefront reconstruction to analyze the full aberrometric profile of the simulated data, establishing a proof of concept for future instrument development. In contrast to prior CNN-based HS approaches that infer aberrations from conventional single-field spot patterns [16,17,18,19], the present work targets multi-eccentric composite HS images acquired in a single exposure, where overlapping/neighboring spot fields from different retinal locations must be robustly reconstructed in parallel.

Our contributions are: (i) a single-shot 3 × 3 multi-eccentric HS sensing concept using one sensor, (ii) a parallel-processing pipeline that returns location-specific aberration estimates, and (iii) a proof-of-concept evaluation on simulated composite HS patterns.

## 2. Materials and Methods

### 2.1. Computational Simulations

The simulations were implemented in MATLAB (R2024a, MathWorks, Natick, MA, USA) using a custom virtual optical model of a HS sensor in which a microlens array formed a spot pattern on the detector plane. Image formation was modeled by propagating the wavefront through the optical system via a generalized pupil function that accounted for pupil size, ocular aberrations, off-axis illumination, and a retinal speckle term.

**Figure 1:**
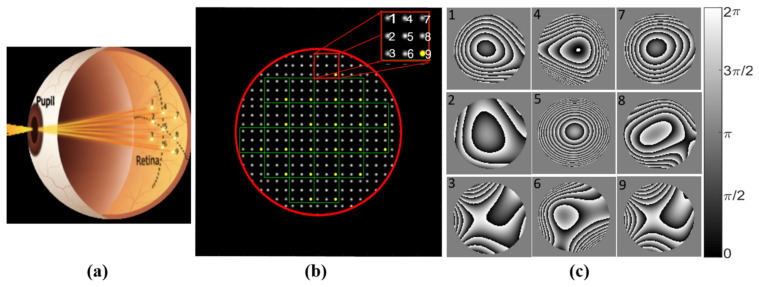
Multi-eccentric Hartmann–Shack (HS) sensor. (**a**) Retinal projection of the nine illumination beams. (**b**) Spot pattern in the HS focal plane showing nine wavefronts recorded simultaneously (yellow-highlighted spots correspond to retinal point 9). (**c**) Reconstructed phase maps for the nine wavefronts.

For multi-eccentric illumination, the concept described in the patent of Suchkov and Wahl [15] was adopted, employing a two-dimensional grating configuration in which one diffraction grating is oriented orthogonally (90°) to the other. This arrangement allowed the incident light beam to be separated into different diffraction orders, generating a 9-point (3 × 3) matrix that systematically covered distinct eccentricities in the visual field, as shown in Figure 1a. The zero-order corresponds to light passing directly through the gratings without deviation and was used to provide the foveal measurement (point 5 in Figure 1a). In contrast, the ±1 orders were set to deviate at angles of ±20° (in the vertical and horizontal direction) relative to the optical axis, enabling measurements in peripheral regions (points 2, 4, 6, 8 at ±20°, and points 1, 3, 7, 9 at ~±27° in Figure 1a).

When these nine rays entered the eye, each was projected onto a specific retinal location, covering both foveal and peripheral regions. The light reflected from each location was then propagated back through the ocular optical system, carrying information characteristic of the local aberrations. The resulting nine wavefronts (each with its corresponding aberrations) were subsequently passed through the microlens array of the HS sensor. In the focal plane of the array, HS images composed of multiple superimposed spot sets were formed (see Figure 1b). Unlike conventional systems in which each microlens produces a single spot, in this configuration each microlens produces a cluster of nine spots—one per eccentricity—yielding a distinctive pattern in the focal plane of the HS sensor. In Figure 1b, a single microlens was zoomed and highlighted with a red box, where the nine spots were identified, each originating from a different wavefront (coming from points 1 to 9 in Figure 1a). Finally, the spot sets corresponding to each wavefront were identified and processed (for example, in Figure 1b, the spots highlighted in yellow correspond to light from point 9 on the retina) and, based on them, nine phase maps were reconstructed, as shown in Figure 1c.

To enable a comprehensive analysis of the proposed system and methodology, two types of simulations were developed: one under ideal optical conditions without speckle effects, and another incorporating speckle, characteristic of the dynamic interaction between coherent light and retinal structures. In addition, two illumination regimes were considered, derived from the non-uniform energy distribution among diffraction orders produced by the gratings, which is determined by the physical characteristics of the simulated phase grating. These characteristics included grating type and profile, and the wavelength of the light used, which was set to 532 nm; in all simulations, two diffraction gratings with a sinusoidal phase profile were employed. This configuration provided a more realistic model of the system behavior under practical conditions.

Firstly, two phase-modulation depths were considered for the simulated gratings: 1 rad and 1.4 rad. The choice of these values was motivated by the dependence of energy distribution among diffraction orders on the modulation depth of a two-dimensional sinusoidal phase grating, i.e., this strategy allows the simulation of two different regimes of illumination in the sensor. This dependence follows from the scalar diffraction theory described by Goodman [20], where the diffraction efficiencies are given by Bessel functions of the first kind; based on this framework, simulations were performed to identify the phase modulation depth that accounts for the homogenization of the spot intensities.

For a modulation of 1 rad, the central order (0, 0—this ordered pair denotes the order in the horizontal and vertical directions, respectively) concentrates approximately 34% of the total energy, while the first-order terms (±1, 0—first order in the horizontal direction) and (0, ±1—first order in the vertical direction) reach around 11% each, and the diagonal orders (±1, ±1) contribute about 4%. Thus, relative to the zero order, each first-order term carries ~32% of the central-order intensity (11/34 ratio), and each diagonal order carries ~12% (4/34 ratio). In contrast, for a modulation of 1.4 rad, the central order (0, 0) concentrates approximately 10% of the total energy; the first-order terms (±1, 0) and (0, ±1) reach around 9% each (~90% of the central-order intensity, 9/10 ratio) and the diagonal orders (±1, ±1) contribute about 8.5% (~85%, 8.5/10 ratio). In this way, two representative conditions are covered: 1 rad, dominated by the central order, and 1.4 rad, reflecting a more balanced redistribution among the first orders—crucial for evaluating performance under different illumination regimes. Because these regimes set the relative spot intensities across eccentricities, they also influence the robustness of spot localization to image degradations such as speckle.

Secondly, in retinal wavefront sensing, coherent illumination produces speckle due to random phase variations introduced by the retinal microstructure. We modeled this effect at the field level by representing the light returning from the retina as a complex wave with a spatially varying random phase superimposed on the aberrated wavefront. Accordingly, for the k-th illumination beam, the pupil-plane field was written as

(1) $$U_{k}\left(r\right)=A_{k}\left(r\right)\times exp\left[i\phi_{k}\left(r\right)+iq_{k}r\right],$$

where $A_{k}\left(r\right)$ is the amplitude, $\phi_{k}\left(r\right)$ is a random phase term associated with retinal scattering, and $q_{k}$ encodes the tilt corresponding to the off-axis (peripheral) illumination angle. Following the fully developed speckle model, the phase screen was generated with $\phi_{k}\left(r\right)\;\sim \;U(0,\;2\pi )$ independently over r, with U treated as a uniform random variable in [0, 2π); the amplitude term $A_{k}\left(r\right)$ was taken as the system’s deterministic pupil and illumination envelope, yielding the negative-exponential intensity statistics characteristic of fully developed speckle. Independent realizations of $\phi_{k}\left(r\right)$ were assigned to different eccentricities to reflect field-dependent retinal texture. After propagation through the ocular optics and microlens array, the sensor records the intensity superposition of the nine spot constellations, producing a composite HS image in which speckle-induced fluctuations bias spot localization and degrade centroid-based reconstruction.

Turning to the parameters of the custom multi-eccentric HS sensor, they were defined from the relationship between dynamic range and the local wavefront slope (see Equation (2)), in which the microlens diameter (*d*) and focal length (*f*) are interdependent and constrained by the maximum local slope. For a maximum slope corresponding to $\theta_{max}=\pm 20{}^{\circ}$, *d* and *f* were selected such that peripheral spots remained within the microlens field.

(2) $$\frac{\partial w(x_{i},y_{i})}{\partial x}=tan\;(\theta_{max})=\frac{d}{2f}.$$

At 20° from the fovea, the nominal peripheral spot (without aberrations) was considered, but additional displacements due to peripheral aberrations also had to be taken into account. If only the nominal position were used, the peripheral spots would fall near microlens edges, limiting the available dynamic range. To estimate the expected aberration-induced displacements, the review by Romashchenko et al. [21]—which compiles key results from multiple studies on peripheral aberrations—was used as a reference. Drawing on data from approximately 2400 eyes, the review reports Zernike coefficients in the periphery (−20° to 20° across the horizontal visual field) within the ranges summarized in Table 1 for a 4 mm-diameter circular pupil. Because defocus dominates the eccentricity-dependent wavefront change and can be adjusted independently in the simulations, it was treated separately from the remaining aberration terms. Treating defocus independently also allows the defocus-driven wavefront-slope contribution to be budgeted explicitly in Equation (2) when selecting d and f, ensuring that both nominal eccentricity offsets and aberration-induced displacements remain within the microlens field. Accordingly, Table 1 summarizes the ranges of the remaining ten Zernike coefficients considered, while the peripheral defocus coefficient $\left(C_{2}^{0}\right)$ was varied independently within ±2 D, consistent with the peripheral defocus values reported in [21] for the −20° to 20° range when considering emmetropic, myopic, and hyperopic eyes. These data were used to statistically characterize the magnitude of peripheral aberrations and to calculate maximum spot displacements, which were added to the base ±20° displacement associated with retinal eccentricity.

As an initial configuration, a microlens array with a pitch of 600 μm and a focal length of 0.60 mm was used to keep spot displacements within the dynamic range of the simulated HS sensor. Simulations were performed for a 4 mm pupil and rendered on a virtual detector of 1000 × 1000 pixels with a 6 μm pixel size, providing sufficient spatial sampling to resolve the multi-eccentric spot clusters. This parameter set was considered as one feasible design within a broader space of possible configurations satisfying the requirements of the proposed multi-eccentric Hartmann–Shack system. Other combinations of microlens pitch, focal length, and detector sampling could also be explored during the optimization of a physical prototype. A future experimental implementation is envisioned by adapting a conventional Hartmann–Shack aberrometer through incorporation of the optical elements required for multi-eccentric illumination, such as the orthogonal diffraction gratings proposed in this work, while preserving the conventional wavefront sensing architecture. This initial choice facilitated reliable identification and separation of the nine-spot patterns produced by multiple wavefronts, which is essential for characterizing peripheral optical aberrations and assessing their impact on image quality. Aberrated wavefronts were synthesized from combinations of Zernike coefficients, with particular emphasis on defocus as described above, and astigmatism, coma, and trefoil drawn within the ranges in Table 1, since these are among the predominant aberrations in human peripheral vision [21]. Because the coefficients in Table 1 are derived from measurements reported along the horizontal visual-field meridian, these ranges were used as representative peripheral aberration ranges for all simulated eccentricities, including the vertical and diagonal field positions, in this controlled computational proof-of-concept evaluation.

### 2.2. Quantification of Aberrations

Wavefront reconstruction was performed by two measurement approaches: (i) a traditional software in which the relative displacements of the spots—between those generated by the aberrated wavefront and their reference position in the absence of aberrations—were integrated; and (ii) a custom convolutional neural network of the ResNet type, designed to estimate and quantify aberrations directly from HS images. In both cases, the reconstructed wavefront was expressed as a Zernike modal expansion up to 4th order (with the simulator allowing inclusion up to 5th order). Following Romashchenko et al. [21], 11 OSA coefficients ($c_{2}^{-2}$ to $c_{4}^{2}$) were considered non-zero for each wavefront.

To analyze the performance of both approaches, images generated by the simulator were processed by each approach under four conditions: ϕ = 1.0 rad without speckle, ϕ = 1.0 rad with speckle, ϕ = 1.4 rad without speckle, and ϕ = 1.4 rad with speckle. For each condition, 5000 HS images were used, yielding 20,000 HS images in total for statistical comparison across speckle and illumination regimes. The same 5000-image split per condition was used as the held-out test set for both the traditional centroid-based pipeline and the ResNet-based approach, allowing performance to be compared on identical data; the implementation details of each pipeline are described next.

In the traditional approach, the compound HS image was first demultiplexed into nine wavefront-specific spot sets by exploiting the known 3 × 3 diffraction-order geometry (see Figure 1). Within each microlens cell, spots were identified and assigned to their corresponding eccentricities according to their nominal relative offsets, yielding nine spot maps (one per wavefront) for subsequent centroid calculations and wavefront reconstruction.

Displacements of the spots produced by local wavefront slopes were measured for each demultiplexed wavefront-specific spot set. The key parameters (pupil diameter, focal length, microlens size) were specified, and the simulated HS image was segmented into a regular matrix of cells, each corresponding to a single microlens. A pyramidal search algorithm was then applied to estimate the centroid (center of mass) within each subcell. This iterative procedure was used to derive the wavefront-gradient map relative to a flat reference, improving robustness under non-uniform intensities and partially truncated spots [22]. Using the gradient map together with the pseudo-inverse of the Zernike derivative matrix evaluated at the microlens positions, the Zernike coefficients were retrieved, and nine reconstructed phase maps (one per wavefront) were generated as the final output.

**Figure 2:**
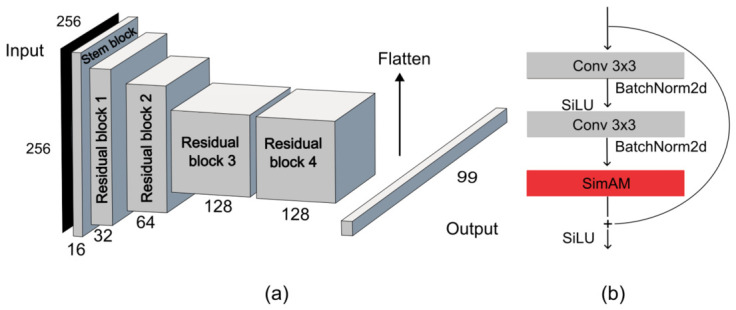
Proposed ResNet–SimAM architecture for direct regression of Zernike coefficients from compound HS images. (**a**) Network overview showing the progression of feature extraction and increasing channel depth across residual stages; a 256 × 256 HS image is mapped to a 99-element output vector (9 wavefronts × 11 coefficients). (**b**) Residual-block structure: stacked 3 × 3 convolutions with batch normalization and SiLU activation, followed by a SimAM attention module before the additive skip connection and a final SiLU non-linearity.

In the learning-based approach, wavefronts were reconstructed with a convolutional neural network configured as an enhanced ResNet with SimAM (Simple, Parameter-Free Attention Mechanism) blocks [23]. In contrast to the centroid-based pipeline, the network was trained to regress the Zernike coefficients directly from the compound HS image, without explicit demultiplexing. The model follows prior demonstrations of deep learning for HS-based wavefront estimation [16,17,18,19,24,25] where other CNN architectures were implemented. The CNN begins with a 3 × 3 convolution (16 filters), batch normalization, and a SiLU (Sigmoid-weighted Linear Unit) activation function, followed by 2 × 2 max pooling for initial feature downsampling (see Figure 2a). This is followed by a hierarchy of four residual blocks with SimAM attention. As shown in the internal layer composition (see Figure 2b), each residual block integrates a processing pipeline of successive 3 × 3 convolutional layers, batch normalization, and SiLU activations, culminating in a SimAM module to modulate feature-channel importance. The channel depth increases progressively, with the first residual block expanding the representation to 32 channels and the second to 64, while subsequent stages reach a depth of 128 channels via strided convolutions. In the final stage, the feature maps are flattened and passed to a fully connected (linear) layer that outputs the predicted Zernike coefficients (99 coefficients—nine wavefronts with 11 coefficients per wavefront in the compound HS image).

Four datasets were prepared, corresponding to the combinations described above for phase modulation (ϕ = 1.0 and 1.4 rad) and speckle (absent/present). For each condition, 76,000 training and 19,000 validation HS images were generated; testing used the shared 5000-image split per condition described earlier in this subsection. A separate model was trained for each dataset. Training was performed in PyTorch (2.5.1, PyTorch Foundation, San Francisco, CA, USA) with the AdamW optimizer (learning rate 1 × 10^−4^), L2 regularization (λ = 1 × 10^−4^), and mean squared error (MSE) as the loss function. To prevent data leakage, HS images derived from the same underlying aberration realization were confined to a single partition (training, validation, or test). Implementation and training were carried out on a workstation equipped with an Intel Core i9-13900KF processor, 64 GB of RAM, and an NVIDIA GeForce RTX 4090 graphics processing unit (GPU).

## 3. Results

### 3.1. Simulated Images

**Figure 3:**
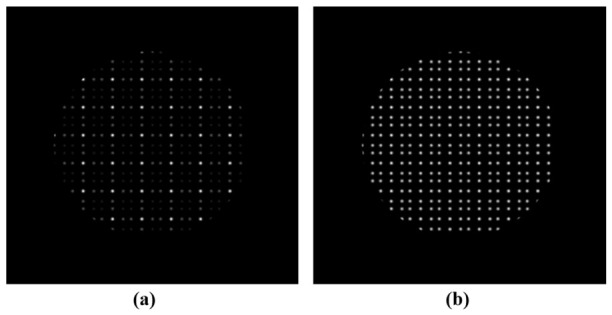
Each image encodes nine wavefronts from peripheral and near foveal retinal points and is rendered without speckle. (**a**) Multi-eccentric HS image with aberrations using a sinusoidal phase-grating modulation ϕ = 1.0 rad. (**b**) Same aberrations as in (**a**) with ϕ = 1.4 rad, yielding a more uniform spot-intensity distribution.

**Figure 4:**
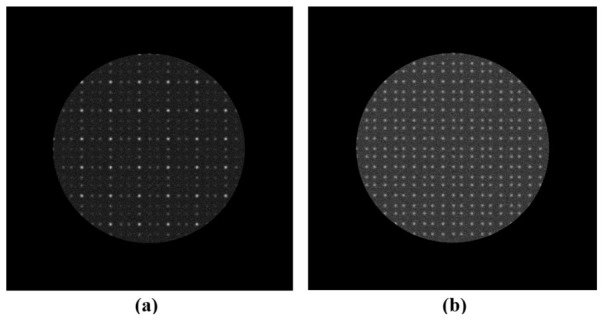
Each image encodes nine wavefronts from peripheral and near foveal retinal points and is rendered with speckle. (**a**) Multi-eccentric HS image with aberrations using a sinusoidal phase grating modulation ϕ = 1.0 rad. (**b**) Same aberrations as in (**a**) with ϕ = 1.4 rad, showing more uniform spot intensities but increased granularity due to speckle.

Figure 3 and Figure 4 illustrate multi-eccentric HS images formed in the focal plane of the simulated microlens array, corresponding to nine wavefronts sampled from retinal locations near ±20° from the fovea.

Without speckle (Figure 3), the influence of the grating phase modulation ϕ on spot intensity is evident. With ϕ = 1.0 rad (Figure 3a), in each microlens, central spots dominate and peripheral spots are dimmer, matching the expected energy distribution. With ϕ = 1.4 rad (Figure 3b), energy is more evenly distributed, yielding more uniform spot intensities within each cell. Identical aberrations were used for the nine wavefronts in both panels.

With speckle included (Figure 4), the same patterns exhibit granular contrast loss and subtle centroid bias due to retinal backscatter. For ϕ = 1.0 rad (Figure 4a), central spots remain relatively brighter but show reduced contrast, and the dimmer peripheral spots lose further definition, complicating centroid estimation. For ϕ = 1.4 rad (Figure 4b), the improved energy balance across the nine spots is maintained, yet all spots display increased granularity. Because each eccentricity contributes its own speckle realization, the superposition of nine speckle patterns accentuates the effect in the final image.

Overall, speckle does not alter the theoretical mean spot positions, but it hampers precise spot identification and thereby reduces reconstruction accuracy for the nine simultaneous wavefronts. These results underscore the need to consider speckle in both instrument design and the processing/filtering pipeline.

### 3.2. Quantification of Aberrations

#### 3.2.1. Traditional Measurement Software

**Figure 5:**
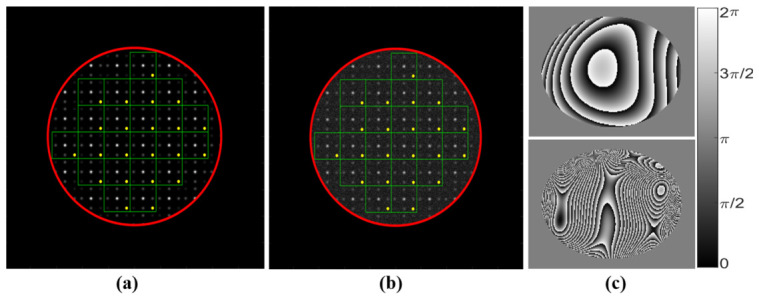
Visualization of spot patterns and a representative phase map for the multi-eccentric HS sensor with ϕ = 1.0 rad (phase modulation of the sinusoidal grating). (**a**) Spot pattern without speckle for nine simultaneously measured wavefronts. (**b**) Spot pattern with speckle, using the same aberrations as in (**a**). (**c**) Phase-map reconstruction for the peripheral position highlighted by yellow markers; the upper map is derived from (**a**) and the lower map from (**b**).

**Figure 6:**
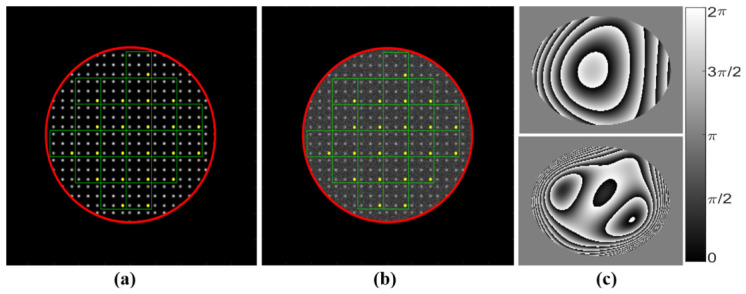
Visualization of spot patterns and a representative phase map for the multi-eccentric HS sensor with ϕ = 1.4 rad (phase modulation of the sinusoidal grating). (**a**) Spot pattern without speckle for nine simultaneously measured wavefronts. (**b**) Spot pattern with speckle, using the same aberrations as in (**a**). (**c**) Phase-map reconstruction for the peripheral position highlighted by yellow markers; the upper map is derived from (**a**) and the lower map from (**b**).

Figure 5 and Figure 6 present representative multi-eccentric HS images. In Figure 5a,b, the spot patterns recorded when nine wavefronts are captured simultaneously with a diffraction grating phase modulation of ϕ = 1.0 rad are shown. Figure 5a depicts the ideal case without speckle, where bright spots appear within the pupil (bounded by the red circle). Figure 5b shows the HS sensor image for the same aberrations but including speckle, characteristic of retinal backscatter. Each green box delimits an individual microlens in the simulated array. Similarly, Figure 6a,b display patterns obtained with ϕ = 1.4 rad, following the same scheme: without speckle in Figure 6a and with speckle in Figure 6b, for identical sets of wavefronts.

Figure 5c and Figure 6c show reconstructed phase maps corresponding to a peripheral location (lower corner), indicated by the yellow markers in panels (a) and (b). In each figure, the upper phase map corresponds to the case without speckle, and the lower map corresponds to the case with speckle.

These results indicate that speckle markedly degrades the accuracy of the traditional algorithm when quantifying Zernike coefficients and reconstructing phase maps from multi-eccentric HS images. Reconstructions under non-ideal conditions (with speckle) exhibit considerable distortions attributable to two main factors: (i) non-uniform energy distribution among diffraction orders, which compromises the interpretation of local wavefront slopes; and (ii) speckle-induced spot morphology changes (irregular shapes and diffuse edges), which hinder precise centroid estimation and thus corrupt slopes and Zernike coefficients retrieval. For ϕ = 1.0 rad (Figure 5c, lower), reconstruction accuracy is lower than for ϕ = 1.4 rad (Figure 6c, lower), consistent with the poorer spot visibility associated with the more uneven illumination at ϕ = 1.0 rad.

Comparison between the upper (no speckle) and lower (with speckle) maps in Figure 5c and Figure 6c further highlights the increased smoothness and regularity achieved under ideal conditions, in contrast to the pronounced irregularities when speckle is present. The magnitude of centroiding error varies with the phase-modulation regime, reinforcing the sensitivity of the traditional pipeline to contrast and spot morphology.

**Figure 7:**
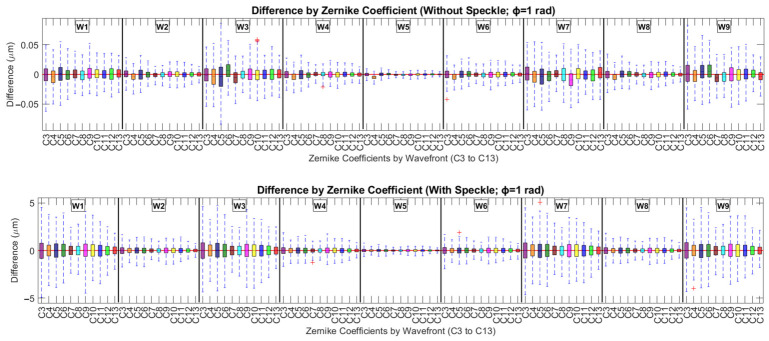
Error in Zernike coefficients (difference: induced minus recovered) for the traditional method, evaluated over 5000 HS images generated with ϕ = 1.0 rad (phase modulation of the sinusoidal grating). The upper panel shows results without speckle, and the lower panel shows results with speckle. W1–W9 denote the nine wavefronts arising from the illuminated retinal points; C3–C13 indicate the Zernike terms (OSA notation).

**Figure 8:**
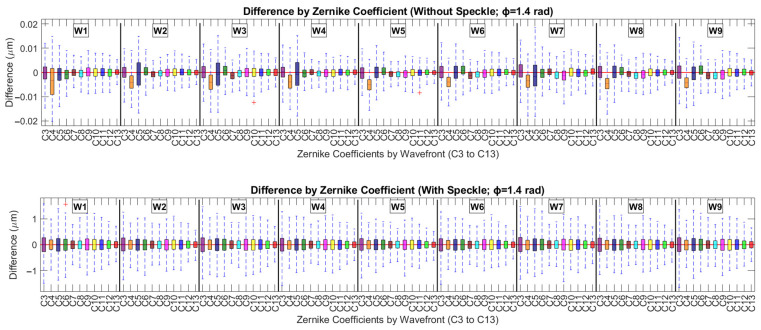
Error in Zernike coefficients (difference: induced minus recovered) for the traditional method, evaluated over 5000 HS images generated with ϕ = 1.4 rad (phase modulation of the sinusoidal grating). The upper panel shows results without speckle, and the lower panel shows results with speckle. W1–W9 index the nine wavefronts arising from the illuminated retinal points; C3–C13 indicate the Zernike terms (OSA notation).

Figure 7 and Figure 8 quantify these effects by plotting the error for each Zernike coefficient, defined as the difference between the coefficient induced in the simulator (from 5000 images per condition with random combinations within the ranges of Table 1) and the value recovered by the traditional method. In Figure 7 (ϕ = 1.0 rad), errors cluster near zero without speckle (upper panel) but show a substantial increase in dispersion and magnitude with speckle (lower panel). Figure 8 shows the corresponding results for ϕ = 1.4 rad: again, the upper panel corresponds to no speckle and the lower to speckle. The higher phase modulation reduces error dispersion relative to Figure 7, even with speckle, owing to the more homogeneous energy distribution and improved spot visibility.

Together, these figures allow direct comparison of the impact of speckle and phase modulation on Zernike-coefficient errors. Speckle notably increases variability and bias, whereas a higher phase modulation (ϕ = 1.4 rad) improves measurement precision by enhancing spot contrast and robustness of the traditional algorithm. The evidence demonstrates critical limitations of the traditional centroid-based pipeline under low or non-uniform illumination (ϕ = 1.0 rad), particularly in the periphery, where phase maps exhibit significant distortions driven by centroid errors on speckled backgrounds. These findings underscore the need for more robust processing strategies and provide a quantitative reference for evaluating and improving future wavefront-analysis algorithms.

#### 3.2.2. ResNet–SimAM Neural Network

Four models based on the ResNet–SimAM architecture were trained using distinct datasets: two models used HS images without speckle at phase modulations ϕ = 1.0 rad and ϕ = 1.4 rad, and two models used HS images with speckle at the same phase modulations. Each model was trained for 50 epochs, with an approximate training time of 30 min per model.

**Figure 9:**
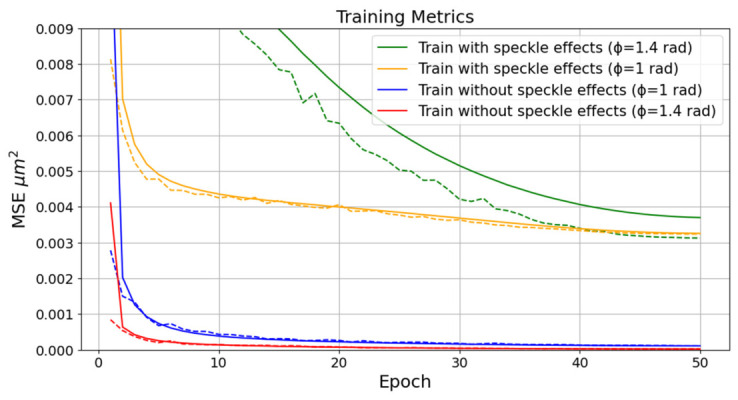
Progression of mean-squared error (MSE) for training (solid lines) and validation (dashed lines) across 50 epochs. Four models are compared: ϕ = 1.0 rad (phase modulation of the sinusoidal grating), no speckle (blue line); ϕ = 1.4 rad, no speckle (red line); ϕ = 1.0 rad, speckle (yellow line); ϕ = 1.4 rad, speckle (green line).

Figure 9 consolidates the training (solid lines) and validation (dashed lines) curves for all four models. Models trained on HS images without speckle (blue and red lines) achieve low MSE and stabilize rapidly near zero, indicating fast convergence and accurate fitting under speckle-free HS images (ideal conditions). In contrast, models trained with speckle noise (yellow and green lines) maintain a higher MSE throughout training, reflecting the added complexity introduced by speckle. Specifically, the ϕ = 1.4 rad model (green lines) exhibits the highest MSE. Notably, the validation curve for this case (dashed green line) settles slightly below the training curve. However, the absolute difference is marginal within this low error regime (<0.001 μm) and is attributed to the high stochastic complexity of these images. The ϕ = 1.0 rad speckle model (yellow lines) shows better performance overall and close correspondence between the training and validation curves. This pattern is consistent across conditions, suggesting good generalization with no evident overfitting.

**Figure 10:**
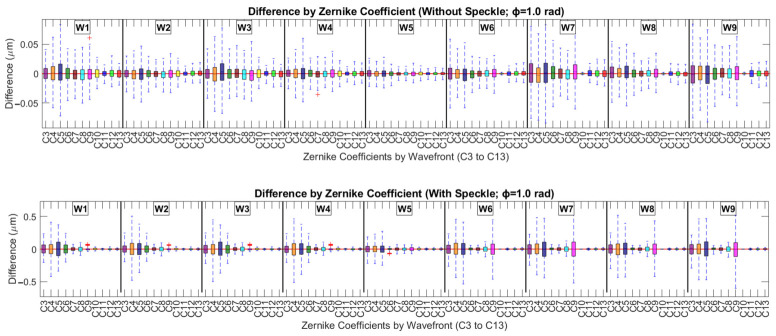
Error in Zernike coefficients (difference: induced minus predicted) for the ResNet–SimAM model, evaluated over 5000 HS images generated with a ϕ = 1.0 rad (phase modulation of the sinusoidal grating). The upper panel shows results without speckle (ideal condition), and the lower panel shows results with speckle. W1–W9 denote the nine wavefronts from the illuminated retinal points; C3–C13 indicate the Zernike terms (OSA notation).

**Figure 11:**
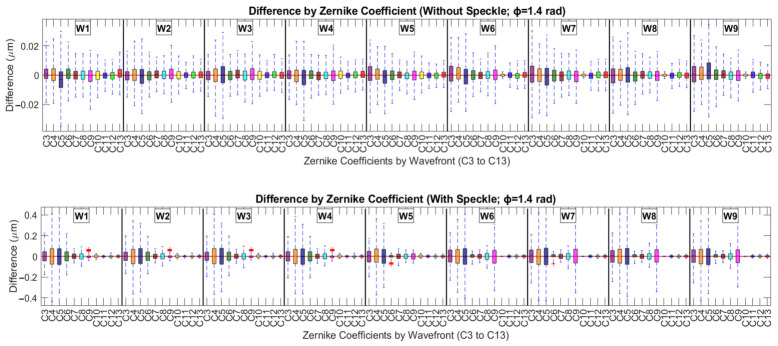
Error in Zernike coefficients (difference: induced minus predicted) for the ResNet–SimAM model, evaluated over 5000 HS images generated with ϕ = 1.4 rad (phase modulation of the sinusoidal grating). The upper panel shows results without speckle (ideal condition), and the lower panel shows results with speckle. W1–W9 denote the nine wavefronts from the illuminated retinal points; C3–C13 indicate the Zernike terms (OSA notation).

To evaluate prediction quality, Figure 10 and Figure 11 report, for each Zernike coefficient, the error defined as the difference between the coefficient induced in the simulator (from 5000 images with random combinations within Table 1 ranges) and the value predicted by the neural network. Figure 10 presents results obtained for ϕ = 1.0 rad: the upper panel corresponds to images without speckle, and the lower panel to images with speckle. In the absence of speckle, the dispersion is considerably smaller (interquartile range ≈ −0.017 μm to 0.018 μm), whereas the presence of speckle increases both the dispersion and the overall range of differences. Nevertheless, values within the box (interquartile range ≈ −0.11 μm to 0.11 μm) remain centered near zero, evidencing robustness to noise. Figure 11 shows the higher-modulation case ϕ = 1.4 rad: again, the upper panel corresponds to images without speckle and the lower panel to images with speckle. Increased phase modulation further reduces both the dispersion and the magnitude of the errors (interquartile range ≈ −0.0081 μm to 0.0078 μm in the absence of speckle, and ≈ −0.079 μm to 0.081 μm in the presence of speckle), demonstrating that the neural network maintains a high level of precision and stability even when speckle is present, and performs better than the traditional method.

A direct comparison between Figure 10 and Figure 11 illustrates the combined effects of speckle and phase modulation on neural network precision, highlighting the network’s superior accuracy in quantifying Zernike coefficients relative to the traditional approach (Figure 7 and Figure 8), especially when speckle is present in the HS images.

## 4. Discussion

As demonstrated by the results, the proposed methodology provides a viable framework for constructing and analyzing images from a multi-eccentric Hartmann–Shack (HS) sensor. The simulations confirm that a single-shot configuration can encode information from nine retinal locations within a single composite HS image, representing a conceptual advance over conventional sequential approaches in which peripheral aberrations are measured point by point.

A second contribution is the generation of composite HS images that reflect key degradations expected in practice. The model incorporates (i) speckle due to coherent retinal reflection and (ii) illumination non-uniformities associated with the diffractive grating, both of which modify spot contrast and intensity across eccentricities. Consequently, the simulated composite image contains nine spot constellations associated with different field positions, with each constellation encoding the local wavefront characteristics (tilt and higher-order aberrations) typical of off-axis measurements. Despite this increased complexity, the spot patterns remain sufficiently structured to support reliable wavefront reconstruction, providing a realistic testbed for algorithmic evaluation.

The selection of retinal speckle and diffraction-order illumination inhomogeneity was motivated by their role as two relevant and complementary sources of degradation in retinal Hartmann–Shack wavefront sensing: one stochastic in nature, the other systematic. Focusing on these two effects enables a controlled evaluation of the reconstruction methods while isolating the influence of representative, mechanistically distinct degradation sources. Although the current simulations do not explicitly model light scattering produced by ocular media opacities, such as incipient cataracts, the proposed computational framework is sufficiently flexible to incorporate additional image degradation mechanisms in future studies, providing a platform for evaluating reconstruction algorithms under more clinically realistic conditions.

Within this framework, the results enable direct and quantitative comparison between a traditional centroid-based reconstruction pipeline and the ResNet–SimAM models for wavefront estimation from simulated multi-eccentric HS images under ideal (speckle-free) and realistic (speckled) conditions. The centroid-based pipeline was selected as the reference method because it represents the conventional reconstruction approach employed in most commercial and research Hartmann–Shack aberrometers, providing a clinically relevant baseline against which to quantify the benefits of the proposed deep-learning approach. Although more advanced classical reconstruction strategies could be explored in future work, this comparison provides insight into the relative robustness of conventional centroiding and learning-based reconstruction when processing composite HS patterns containing information from multiple retinal eccentricities.

Figure 7 and Figure 8 show that the traditional software is highly sensitive to speckle, as expected [26]. In the absence of speckle, Zernike coefficient errors remain low—approximately ±0.05 µm for ϕ = 1.0 rad (phase modulation of the sinusoidal grating) and ±0.02 µm for ϕ = 1.4 rad (upper panels). When speckle is present, dispersion and magnitude increase markedly, reaching up to ±5 µm for ϕ = 1.0 rad and around ±1 µm for ϕ = 1.4 rad (lower panels). Sensitivity is accentuated at the lower phase modulation (ϕ = 1.0 rad), indicating strong dependence on spot visibility and definition. The combined effect of speckle and reduced peripheral spots energy within each cell compromises centroid detection and, consequently, reconstruction accuracy. Nevertheless, it is noteworthy that the implemented centroid-pyramidal search retrieves nine simultaneous wavefronts coming from a single HS multi-eccentric image—to the best of our knowledge, an initial demonstration of nine-wavefront reconstruction from a single composite HS image.

By contrast, Figure 10 and Figure 11 indicate that the CNN models maintain tightly constrained errors around zero under both speckle-free and speckled conditions. For ϕ = 1.0 rad, differences between induced and predicted Zernike coefficients remain within approximately ±0.02 µm without speckle and increase to about ±0.2 µm with speckle. For ϕ = 1.4 rad, the ranges are reduced further—below ±0.01 µm (ideal) and around ±0.1 µm (speckled). These magnitudes are one to two orders of magnitude smaller than those obtained with the traditional method (±1 and ±5 µm under similar conditions), confirming superior robustness against speckle-induced distortions and illumination inhomogeneities. Additionally, higher phase modulation (ϕ = 1.4 rad) improves overall precision for both methods by balancing energy among diffraction orders and enhancing spot contrast. The CNN models also exhibit consistently lower variability across all nine wavefronts (W1–W9), supporting greater stability and accuracy under realistic optical conditions.

Table 2 summarizes the quantitative comparison between the ResNet–SimAM model and the traditional centroid-based software under speckled and speckle-free conditions for ϕ = 1.0 rad and ϕ = 1.4 rad (phase modulation of the sinusoidal grating), with all metrics averaged across the nine multi-eccentric wavefronts (W1–W9). For each method, the correlation coefficient (*cr*) quantifies the linear association between the reference and estimated Zernike coefficients, whereas the root-mean-square error (RMSE), defined as the square root of the mean squared difference between them, quantifies the magnitude of the residual error. Under ideal (speckle-free) conditions, both methods show high correlation with the reference coefficients; however, ResNet–SimAM achieves correlation closer to unity (cr ≈ 0.92–0.97) and consistently low RMSE values (≤0.01 μm). In the presence of speckle, the traditional method exhibits a pronounced loss of accuracy, with correlations dropping to cr ≈ 0.17–0.23 and RMSE values reaching up to 0.55 μm, whereas ResNet–SimAM maintains moderate correlations (cr ≈ 0.76–0.78) and low RMSEs (≈0.05 μm).

These results quantitatively confirm the trends observed in Figure 10 and Figure 11: the neural-network model consistently preserves accuracy and stability across different modulation depths and noise levels, clearly outperforming the traditional centroid-based algorithm under realistic optical conditions.

**Figure 12:**
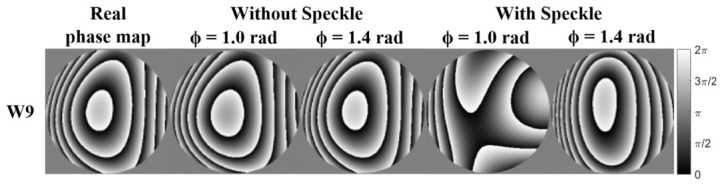
Reconstructed phase maps for wavefront W9, obtained from the Zernike coefficients predicted by the ResNet–SimAM model. The CNN-based method preserves the main structural features of the original wavefront and provides smoother, more faithful reconstructions than the traditional centroid-based approach, even in the presence of speckle.

Visual inspection of reconstructed phase maps (Figure 5c and Figure 6c) corroborates these trends. Under speckle-free conditions, both phase modulations yield reconstructions that are consistent with the induced wavefronts. With speckle, the traditional method produces phase maps with prominent distortions, particularly in the periphery. In contrast, when the Zernike coefficients predicted by the CNN models are used for reconstruction, the principal features of the original wavefront are preserved. Figure 12 shows an example of the reconstructed phase map for wavefront W9 and the complete set of maps (W1–W9) is presented in Figure S1 of the Supplementary Material, evidencing higher fidelity—especially for HS images with speckle.

**Figure 13:**
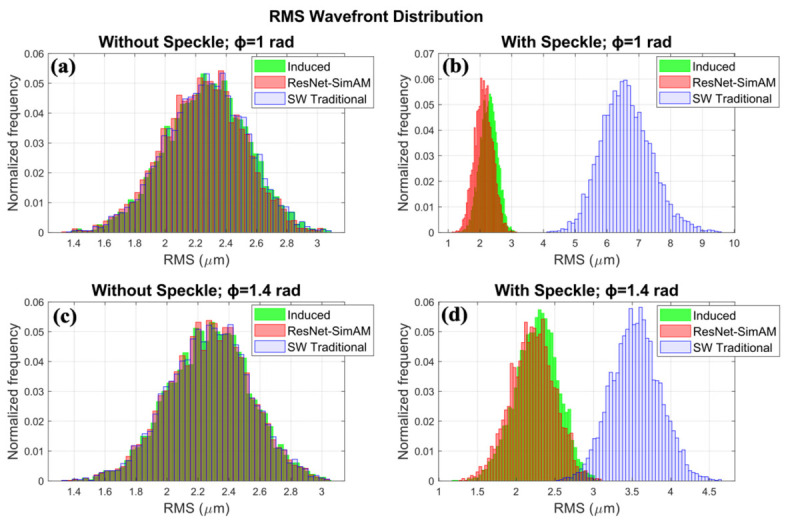
Comparison of RMS wavefront distributions for the traditional centroid-based software (blue bars), the ResNet–SimAM model (red bars), and the induced/reference values (green bars). Distributions (histograms) are computed from the same 5000 simulated HS images per condition (random Zernike coefficients). Panels (**a**,**c**) present speckle-free cases for ϕ = 1.0 rad and ϕ = 1.4 rad (phase modulation of the sinusoidal grating), respectively. Panels (**b**,**d**) present the corresponding speckled cases.

Figure 13 presents the root-mean-square (RMS) wavefront distributions for the traditional method (blue bars), the ResNet–SimAM model (red bars) and the induced (reference) values (green bars). The RMS wavefront value, computed as the square root of the sum of the squared Zernike coefficients, provides a compact measure of the overall aberration magnitude of each wavefront. The distributions are obtained from the same 5000 simulated HS images per condition, corresponding to 45,000 reconstructed wavefronts (5000 compound HS images × 9 wavefronts per image); panels (a,c) show speckle-free results for ϕ = 1.0 and ϕ = 1.4 rad, and panels (b,d) show the corresponding speckled cases. Under ideal conditions (Figure 13a,c), both methods reproduce the reference distribution closely, as indicated by the overlap of blue (traditional), red (ResNet–SimAM), and green (reference) histograms, regardless of ϕ. Under realistic conditions (Figure 13b,d), the ResNet–SimAM model remains aligned with the reference RMS distributions across modulations depths, whereas the traditional software shifts toward higher RMS errors, indicating a consistent loss of precision when spot intensities are non-uniform and speckle is present.

Overall, while both approaches perform well under ideal conditions, the neural network is markedly more robust to degradations in image quality, such as speckle or low peripheral illumination. This robustness translates into more precise wavefront reconstruction and more stable Zernike-coefficient estimates with the ResNet–SimAM models under conditions that better mimic the human ocular system.

In summary, extending centroid-based analysis to simultaneous multi-eccentric measurements increases sensitivity to energy redistribution and speckle, which reduces spot contrast and degrades localization. The neural-network approach proved more robust under realistic conditions, maintaining stable reconstruction across wavefronts despite intensity inhomogeneities. These results support the feasibility of single-shot multi-eccentric HS sensing as a foundation for future physical instrument development.

These advances open opportunities to investigate peripheral aberrations with greater precision and to assess treatments and devices designed to address peripheral defocus (e.g., MiSight, Stellest, Miyosmart [27]). The approach lays the groundwork for developing physical instruments and more effective clinical strategies, and it illustrates how artificial intelligence can increase robustness when composite HS patterns are degraded by speckle and non-uniform illumination. Overall, the work demonstrates the feasibility of single-shot 3 × 3 multi-eccentric HS sensing with a single sensor and provides a proof-of-concept pipeline for extracting location-specific aberration estimates from composite measurements.

## 5. Conclusions

We presented a computational simulation of a multi-eccentric Hartmann–Shack aberrometer, inspired by the patent of Suchkov and Wahl [15], that captures nine wavefronts in a single shot at peripheral retinal locations. Simulations under ideal (speckle-free) and realistic (with speckle and illumination inhomogeneities) conditions enabled a comparative evaluation of two reconstruction pipelines under identical conditions: a centroid-based traditional method and a ResNet–SimAM convolutional neural network.

Under ideal conditions, the traditional method reconstructs wavefronts accurately. In realistic conditions, however, speckle and non-uniform spot intensities degrade centroid estimates and increase Zernike-coefficient errors—particularly at lower phase modulation, where peripheral spots are dimmer. The ResNet–SimAM approach remains robust across conditions, delivering smoother phase-map reconstructions and substantially lower coefficient errors. Training on speckled images further improves resilience by learning the image-level effects of speckle (contrast loss, granularity, centroid bias) encoded in the training images.

These findings demonstrate the potential of the proposed framework as a computational proof of concept for simultaneous multi-eccentric wavefront sensing and motivate the development of a physical multi-eccentric Hartmann–Shack (HS) prototype for peripheral aberrometry, with particular relevance to evaluating optical strategies intended to control peripheral defocus. The proposed framework also provides a flexible computational platform for the objective evaluation and comparison of wavefront reconstruction algorithms under controlled multi-eccentric imaging conditions prior to experimental implementation.

Future work will focus on the implementation, calibration, and experimental validation of the proposed system, including the quantification of practical constraints such as exposure time, fixation instability, and eye motion during acquisition, with reconstruction accuracy validated against experimental measurements across eccentricities. The computational framework can also be extended to incorporate additional clinically relevant degradation mechanisms, enabling a platform for evaluating reconstruction algorithms under increasingly realistic imaging conditions.

## Figures and Tables

**Table 1:** Peripheral Zernike coefficients (astigmatism and higher-order terms; defocus $C_{2}^{0}$ excluded) across the horizontal visual field (reported in μm, 4 mm circular pupil) from Romashchenko et al. [21]. Negative angles correspond to the temporal field and positive angles to the nasal field. Entries are population mean ± standard deviation. The defocus term was not included in this table because it was simulated separately over ±2 D (see text).

Zernike Coefficients	−20°	0°	20°
$C_{2}^{-2}$	0.035 ± 0.136	−0.038 ± 0.125	−0.047 ± 0.154
$C_{2}^{2}$	0.030 ± 0.239	−0.030 ± 0.223	0.296 ± 0.349
$C_{3}^{-3}$	−0.009 ± 0.040	−0.019 ± 0.044	−0.007 ± 0.037
$C_{3}^{-1}$	0.015 ± 0.046	0.007 ± 0.044	0.001 ± 0.038
$C_{3}^{1}$	0.108 ± 0.066	0.006 ± 0.041	−0.125 ± 0.076
$C_{3}^{3}$	0.012 ± 0.039	0.000 ± 0.036	−0.018 ± 0.405
$C_{4}^{-4}$	0.002 ± 0.016	0.002 ± 0.015	0.002 ± 0.011
$C_{4}^{-2}$	−0.001 ± 0.008	0.000 ± 0.009	0.000 ± 0.009
$C_{4}^{0}$	0.009 ± 0.020	0.015 ± 0.020	0.016 ± 0.019
$C_{4}^{2}$	0.000 ± 0.011	0.000 ± 0.012	0.002 ± 0.013

**Table 2:** Quantitative comparison between the ResNet–SimAM model and the traditional centroid-based software (SW Tradicional) for predicting Zernike coefficients under different phase-modulation conditions (ϕ) and speckle presence. The correlation coefficient (cr) quantifies the linear association between the reference Zernike coefficients and those estimated by each reconstruction method, and the root-mean-square error (RMSE) is computed from the differences between the reference and estimated coefficients. All values are averaged over the nine multi-eccentric wavefronts (W1–W9).

	Without Speckle				With Speckle			
	ResNet–SimAM		SW Tradicional		ResNet–SimAM		SW Tradicional	
ϕ	cr	RMSE	cr	RMSE	cr	RMSE	cr	RMSE
1.0	0.92	0.015	0.87	0.009	0.76	0.052	0.17	0.55
1.4	0.97	0.007	0.96	0.003	0.78	0.046	0.23	0.27

## Data Availability

The synthetic datasets and MATLAB scripts used to generate the simulated multi-eccentric Hartmann–Shack images supporting the findings of this study are publicly available in a Zenodo repository at https://doi.org/10.5281/zenodo.21227985.

## Supplementary Materials

The following supporting information can be downloaded at https://www.mdpi.com/article/10.3390/s26144419/s1, Figure S1. Examples of phase maps reconstructed from Zernike coefficients predicted by the ResNet-SimAM model for the nine wavefronts (W1–W9) of a multi-eccentric HS image. The CNN-based method preserves the main features of the induced wavefronts and yields smooth, faithful reconstructions even when the HS images include speckle.

## Abbreviations

The following abbreviations are used in this manuscript:

HS	Hartmann–Shack
SW	Software
CNN	Convolutional Neural Networks
SimAM	Simple, parameter-free Attention Mechanism
SiLU	Sigmoid-weighted Linear Unit
W	Wavefronts
cr	Correlation
MSE	Mean Squared Error
RMS	Root-Mean-Square
RMSE	Root-Mean-Square Error
