# Supplementary material for: Simultaneous Measurement of Peripheral Ocular Aberrations Using a Virtual Multi-Eccentric Hartmann–Shack Aberrometer
Source: Sensors (Basel). 2026 Jul 12;26(14):4419. doi: 10.3390/s26144419 (PMC13418118; doi:10.3390/s26144419)
Supplement: Supplementary file 1 [file sensors-26-04419-s001.zip › sensors-4291771-supplementary.pdf]

Supplementary Material

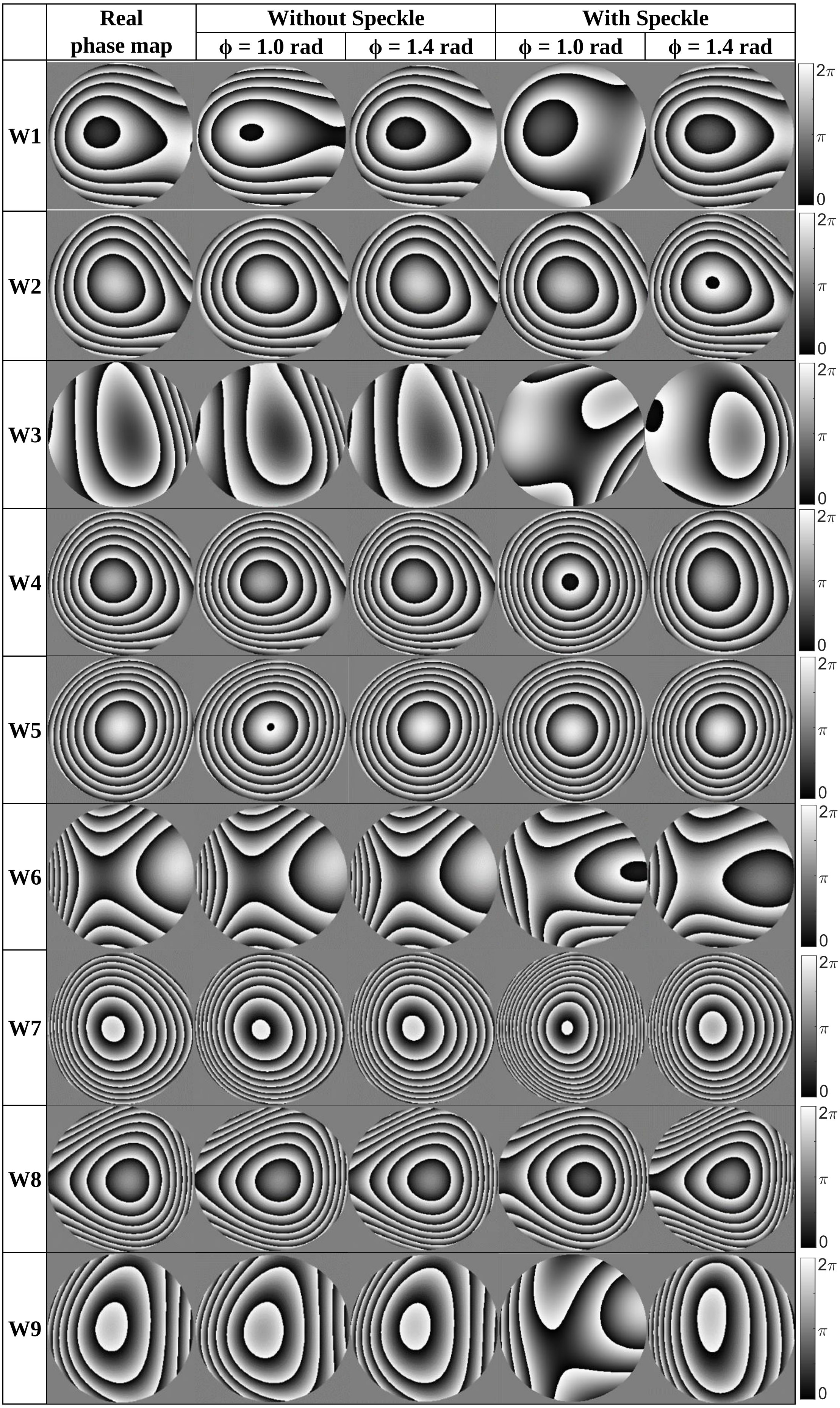

Figure S1. Examples of phase maps reconstructed from Zernike coefficients predicted by the ResNet-SimAM model for the nine wavefronts (W1–W9) of a multi-eccentric HS image. The CNN-based method preserves the main features of the induced wavefronts and yields smooth, faithful reconstructions even when the HS images include speckle.
